# Physical and Rehabilitation Therapy for Overactive Bladder in Women: A Systematic Review and Meta-Analysis

**DOI:** 10.1155/2023/6758454

**Published:** 2023-01-04

**Authors:** Jingwen Bai, Yilan Tian, Yiran Wang, Xiaofang Zhang, Ping Wang

**Affiliations:** ^1^Department of Obstetrics and Gynecology, The West China Second University Hospital, Sichuan University, No. 20 Section Three, South Renmin Road, Chengdu 610041, Sichuan Province, China; ^2^Department of Obstetrics and Gynecology, Affiliated Hospital of Chengdu University, Chengdu 610081, Sichuan Province, China

## Abstract

**Objective:**

To compare the effects of different physical and rehabilitation therapies on women with overactive bladder (OAB).

**Design:**

Network meta-analysis. Data source: The Embase, Scopus, and PubMed databases were systematically searched from their inception to June 22, 2022. We included only RCTs, with no language restrictions. Articles in the reference lists and related studies were thoroughly reviewed. *Review Methods*. This network meta-analysis included related studies on different physical and rehabilitation therapies for OAB. Data were extracted independently from the included randomized controlled trials by two authors, and they used the Cochrane Collaboration's tool to evaluate the risk of bias. We used RevMan to assess the risk assessment of research bias. This network meta-analysis was performed using the Stata software. We completed the review in accordance with the PRISMA items for systematic reviews and meta-analyses statement.

**Results:**

Twelve randomized controlled trials involving 637 patients were included in this meta-analysis. All physical and rehabilitation therapies improved daytime micturition frequency and nocturia frequency in OAB patients. Percutaneous tibial nerve stimulation (PTNS), BT + ES, and BT + BF + ES are better interventions for OAB treatment. There were no significant differences in PTNS, BT + ES, and BT + BF + ES.

**Conclusion:**

All physical and rehabilitation therapies can improve daytime micturition and nocturia frequency in OAB. PTNS, BT + ES, and BT + BF + ES were the priority therapies.

## 1. Introduction

Overactive bladder (OAB) is defined by the International Continental Society (ICS) as urgency, with or without urge incontinence, usually with frequency and nocturia, which is a symptom syndrome [1]. Diseases with definite pathological factors or those associated with infection must be excluded from the diagnosis [1]. The reported incidence among females in different regions varies, with approximately 6% incidence in China [2], 16.9% in the United States [3], and 16.6% in Europe [4], and an overall trend that increases with age [5, 6]. Moreover, OAB has a significant negative impact on the quality of live, also with increasing healthcare costs [7–10].

The American Urology Association and Society of Urodynamics, Female Pelvic Medicine, and Urogenital Reconstruction guidelines recommend four levels of management for OAB: conservative therapy, drug therapy, intradetrusor injection of onabotulinum toxin, sacral neuromodulation, and peripheral tibial nerve stimulation, and surgical therapy [11]. Among the therapeutic techniques for OAB, drug therapy is the simplest and most convenient, which can effectively relieve the symptoms of OAB [12]. However, due to side effects such as dry mouth, constipation, and vision irregularity, the sustainability of drug therapy is seriously affected [13, 14]. Surgical treatment is often used after failure of drugs, physical therapy, and other therapies [15, 16].

Physical and rehabilitation therapies provide more options for female patients with OAB. However, there is a lack of relevant comparative research. Therefore, we searched for relevant randomized controlled trials (RCTs) to compare the effects of different physical and rehabilitation therapies in women with OAB.

## 2. Materials and Methods

### 2.1. Search Strategy

We performed a network meta-analysis of all studies according to the PRISMA 2020 statement [17]. The Embase, Scopus, and PubMed databases were systematically searched from their inception to June 22, 2022. We searched for titles, abstracts, and keywords by combining the following terms: (overactive bladder) OR (overactive detrusor)) AND (bladder training) OR (pelvic floor muscle training) OR (biofeedback) OR (electric^∗^) OR (Kinesio taping) OR (pelvic floor muscle exercise) OR (PTNS) OR (percutaneous tibial nerve stimulation) OR (TTNS) OR (transcutaneous tibial nerve stimulation) OR (tibial nerve stimulation) OR (sacral neuromodulation) AND (random^∗^). There were no language restrictions. Articles in the reference lists and related studies were thoroughly reviewed. We did not register for this study.

### 2.2. Inclusion and Exclusion Criteria

Through a systematic review of all relevant literature, the following studies that met the criteria were included: (1) randomized controlled trials of physical and rehabilitation therapy in women with OAB and (2) women over the age of 18 years. Studies that met the following criteria were excluded: (1) studies of drug therapy or studies without drug washout; (2) studies with OAB surgery; (3) infectious factors, such as urinary tract infection or other obvious pathological changes, such as neurological diseases; and (4) other literature, such as conference abstracts, systematic reviews or meta-analyses, case reports, letters to the editor, or comments.

### 2.3. Data Extraction

All data were extracted by two authors from randomized controlled trials that met the inclusion criteria. The year of publication, specific randomization method, sample size of patients, interventions, and outcomes after the intervention were gathered from each study. We entered the extracted information into Microsoft Excel, and any conflict between the two authors was resolved through full discussion with the third author.

### 2.4. Risk of Bias Assessment

The risk of bias assessment for the included trials was performed independently by two authors using the Cochrane Collaboration's tool [18]. Factors included in the evaluation were sequence generation, allocation concealment, blinding, incomplete outcome data, selective outcome reporting, and other issues. Any disagreement during the evaluation was resolved through a full discussion with the third author.

### 2.5. Statistical Analysis

Review Manager Software (RevMan 5.3; Cochrane Corporation) was used to assess the risk of research bias [19]. The pairwise meta-analysis of direct comparisons was performed using STATA-17.0 (StataCorp, Texas). The mean difference (MD) and 95% confidence interval (CIs) were used to describe the results for continuous data. Statistical significance was set at *P* < 0.05. Statistical heterogeneity among studies was assessed using the *I*^2^ statistic. If *I*^2^ ≤ 50%, a fixed-effects model was used for synthesis, otherwise, the random effects model was used. Subgroup and sensitivity analyses were performed when necessary. We used STATA-17.0's “network map,” “ifPlot,” “netfunnel,” and “sucra” programs to generate the resulting statistical graphs.

## 3. Results

### 3.1. Identification of Relevant Studies

**Figure 1:**
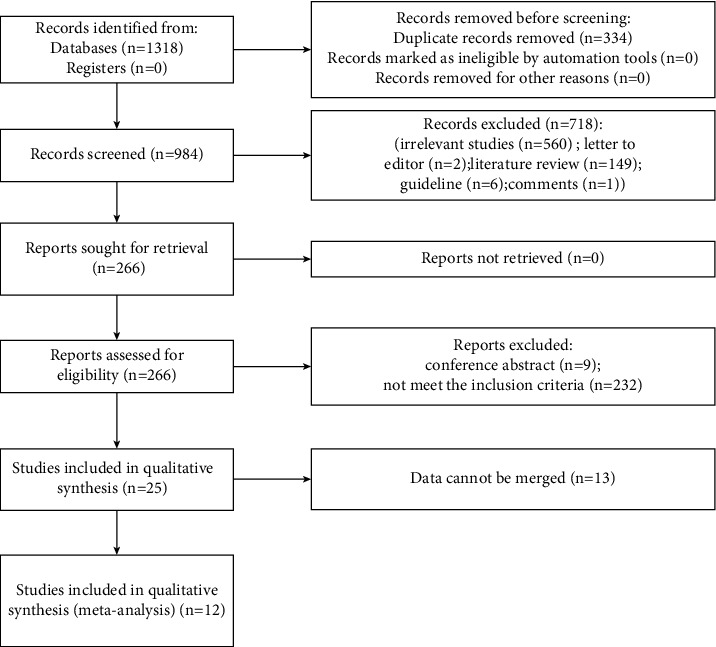
Flow diagram of study selection.

We identified 1318 studies by searching the Embase, Scopus, and PubMed databases. Of these, 334 duplicate studies were deleted, and 984 studies were retrieved, with a total of 266 randomized controlled trials. Among them, nine were conference abstracts, and 232 were deleted according to the exclusion criteria. Finally, 25 studies were included according to the inclusion criteria. The risk of literature bias was assessed based on the results reported by the studies, and data from only 12 studies were combined for this network meta-analysis. A research flowchart related to this literature review is shown in Figure 1.

### 3.2. Characteristics of the Included Studies and Risk of Bias Assessment

**Figure 2:**
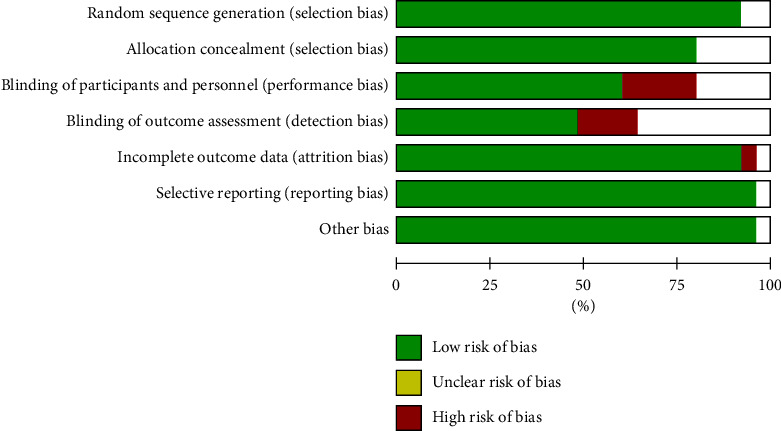
Risk of bias graph.

**Figure 3:**
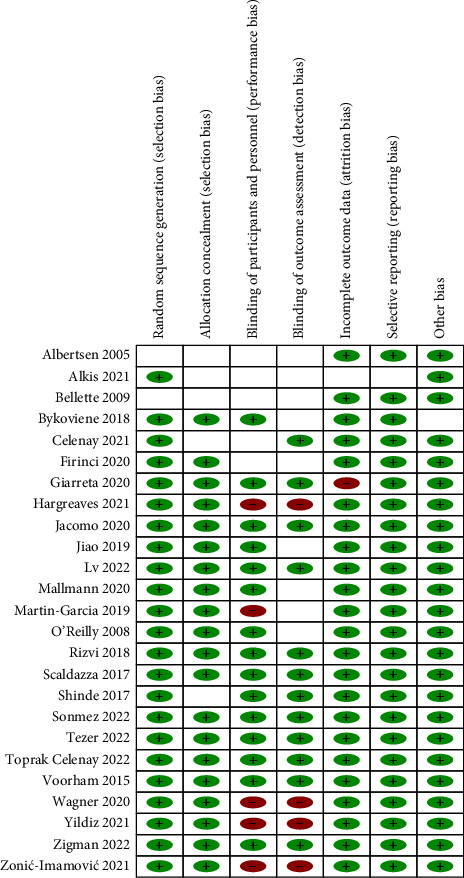
Risk of bias summary.

For the 25 RCTs included, the risk of bias was assessed using RevMan 5.3. The generated risk of bias graph is shown in Figure 2, and the risk of bias summary is shown in Figure 3. In terms of random sequence generation, 92% had a low risk of bias and 8% had an unclear risk of bias. In terms of allocation concealment, 80% had a low risk of bias and 20% had an unclear risk of bias. In terms of blinding of participants and personnel, 60% had a low risk of bias, 20% had a high risk of bias, and 20% had an unclear risk of bias. In terms of blinding of outcome assessment, 48% had a low risk of bias, 16% had a high risk of bias, and 36% had an unclear risk of bias. In terms of incomplete outcome data, 92% had a low risk of bias, 4% had a high risk of bias, and 4% had an unclear risk of bias. In terms of selective reporting, 96% had a low risk of bias and 4% had an unclear risk of bias. In terms of other biases, 96% had a low risk of bias, and 4% had an unclear risk of bias.

**Figure 4:**
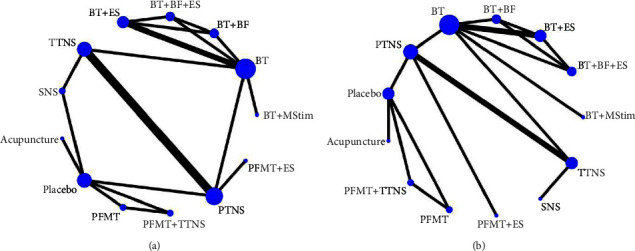
Network of the comparisons for the Bayesian network meta-analysis. (a) Network of the comparisons for the daytime micturition frequency. (b) Network of the comparisons for the nocturia frequency.

Among the 25 studies, only 12 studies collected data on daytime micturition frequency or nocturia frequency; therefore, 12 studies were included in the meta-analysis. A total of 637 women completed the 12 randomized control trials in this network meta-analysis. Twelve physical and rehabilitation therapies for OAB were included.  Table 1 summarizes the basic characteristics of the included studies. There were eight two-arm trials, two three-arm trials, and one four-arm trial. A total of 112 patients were treated with percutaneous tibial nerve stimulation (PTNS), 20 with pelvic floor muscle training (PFMT), 87 with transcutaneous stimulation of the tibial nerve (TTNS), 19 with PFMT + TTNS, and 30 with PFMT + electrical stimulation (ES). A total of 101 patients were treated with bladder training (BT), 16 with BT + biofeedback (BF), 46 with BT + ES, 16 with BT + BF + ES, 16 with acupuncture, 58 with sacral nerve stimulation (SNS), and 35 with BT + magnetic stimulation (MStim).  Figure 4 shows the “network map” of daytime micturition and nocturia frequencies.

### 3.3. Inconsistency Test

**Figure 5:**
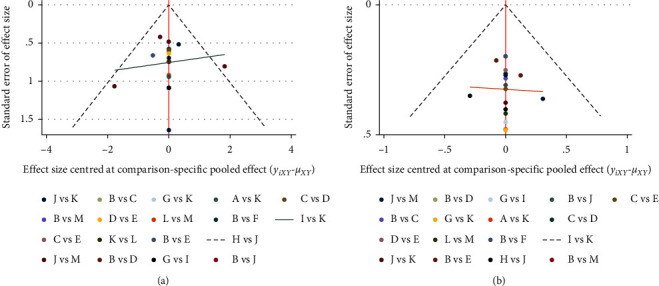
Funnel plot of this network meta-analysis. (a) Funnel plot of the daytime micturition frequency. (b) Funnel plot of the nocturia frequency.

**Figure 6:**
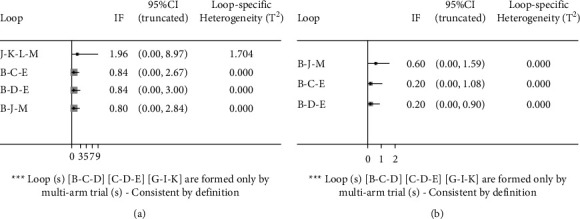
Inconsistency plot of this network meta-analysis. (a) Inconsistency plot of the daytime micturition frequency. (b) Inconsistency plot of the nocturia frequency.

The comparison-corrected funnel plots for daytime micturition and nocturia frequencies are shown in Figure 5. All the studies were symmetrically distributed on both sides of the midline in the middle and upper parts, suggesting a low risk of publication bias. Inconsistencies were tested for daytime micturition frequency and nocturia frequency, with *P* values of 0.9680 and 0.3628, respectively; hence, there were no inconsistencies observed. As shown in Figure 6, the ring inconsistency detection shows that the ring inconsistency is not significant, and the direct comparison results are basically consistent with the indirect comparison results; therefore, it cannot be considered that there is great heterogeneity.

## 4. Meta-Analysis of Physical and Rehabilitation Therapy for Overactive Bladder

### 4.1. Daytime Micturition Frequency

**Figure 7:**
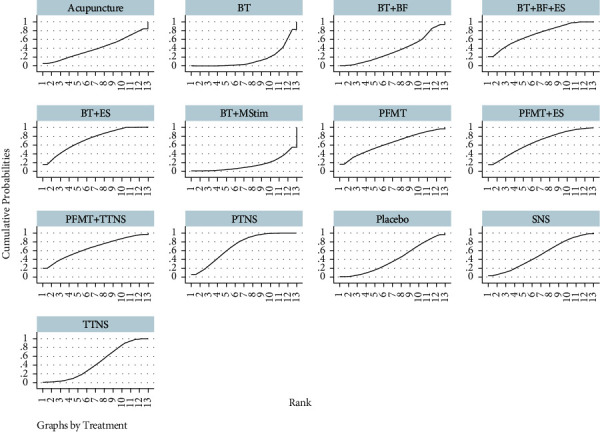
Cumulative ranking curves for the daytime micturition frequency.

A Bayesian mesh meta-analysis of the daytime micturition frequency was conducted for the included studies. The SUCRA values are shown in Figure 7, and the probability ranking results show that the advantages and disadvantages of different interventions are ranked in the following order: BT + ES >  BT + BF + ES > PTNS > PFMT + TTNS > PFMT + ES >  PFMT >  SNS > TTNS > Placebo > Acupuncture > BT + BF > BT > BT + MStim. There were 78 pairwise comparisons in the network meta-analysis, of which, four were statistically significant (*P* < 0.05). BT + ES, BT + BF + ES, and PTNS were superior to BT alone. BT + ES were superior to BT + MStim, and there were no significant differences among the other interventions.

### 4.2. Nocturia Frequency

**Figure 8:**
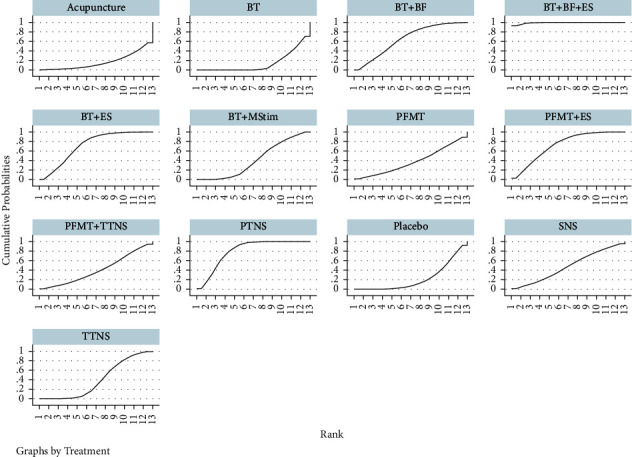
Cumulative ranking curves for nocturia frequency.

A Bayesian mesh meta-analysis of nocturia frequency was conducted on the included studies. The SUCRA values are shown in Figure 8, and the probability ranking results show that the advantages and disadvantages of different interventions were ranked in the following order: BT + BF + ES > PTNS > PFMT + ES  >  BT  +  ES  >  BT  +  BF  >  SNS >  BT + MStim > PFMT + TTNS > TTNS > PFMT > placebo > acupuncture > BT, with 78 pairwise comparisons in the network meta-analysis, and 18 of which were statistically significant (*P* < 0.05). BT + BF + ES were superior to 11 treatments, except PFMT + ES; PTNS was superior to TTNS, placebo, acupuncture, and BT; PFMT + ES, BT + ES, and BT + BF were superior to BT, and there were no significant differences among the other interventions.

## 5. Discussion

Physical and rehabilitation therapies for OAB are either single or combined. According to this network meta-analysis, all therapies can reduce the daytime micturition frequency or nocturia frequency of OAB to a certain extent. Combined with the improvement in daytime micturition frequency and nocturia frequency, PTNS in single therapy and BT + ES and BT + BF + ES in combination therapy may be effective therapies for OAB treatment. There was no significant difference in PTNS, BT + ES, and BT + BF + ES.

Bladder training (BT), as the first-line recommended therapy for female incontinence can reduce the frequency of urination [20], which is consistent with the results of this network meta-analysis. However, only BT can improve the frequency of urination to a limited extent; therefore, Firinci et al., Rizvi et al., and Phillips et al. tried to find a better combination therapy based on BT [21–23]. The results of this network meta-analysis showed that BT + ES and BT + BF + ES showed better therapeutic effects in controlling daytime micturition frequency and nocturia frequency, and that aging leads to a reduction in bladder and urethral function [6, 24, 25]. RCT studies included in this network meta-analysis used transvaginal electrodes for electrical stimulation therapy, which can effectively reduce pudendal nerve afferents or inhibit detrusor activity through current stimulation [26, 27], thus improving OAB symptoms. In these two RCT studies, ES was treated three days a week with a single treatment time of 20 minutes for 24 times, i.e., the total treatment time was 8 weeks. ES therapy settings were as follows: 10 HZ stimulation parameters, work-rest cycle 5–10 s, pulse width 100 ms. Symmetrical biphasic pulses were adjusted between 1 and 100 mA depending on the patient's discomfort level [4, 21]. Based on the improvement in daytime micturition frequency and nocturia frequency, this setting seems reasonable. BT + ES can effectively reduce the daytime micturition frequency and nocturia frequency, patients with ES therapy have good tolerance, and adverse reactions include discomfort and vaginal infection [4, 28]. Whether there is a better setting method for electrical stimulation and therapy course needs further attention in future studies, as it can guide patients to correct contraction control of pelvic floor muscle activity, thus making pelvic floor muscle training more effective [29]. As seen from the RANK plot, BT + BF + ES is the best therapy for improving nocturia frequency and one of the best interventions to improve daytime micturition frequency. In practice, biofeedback can be used at the same time as ES, and the treatment time needs to be extended each time, with good acceptance by patients [21]. Percutaneous tibial nerve stimulation (PTNS), which is a minimally invasive treatment, uses a fine needle inserted 5 cm cephalad from the medial malleolus and posterior to the edge of the tibia [30, 31]. The sacral plexus (S2–S4) is stimulated to reach the sacral urination center by stimulating the afferents of the tibial nerve (which contains the parasympathetic plexus of the bladder), and detrusor relaxation is induced by the inhibition of parasympathetic motor neurons [30, 32]. Because PTNS is a minimally invasive treatment, some scholars have proposed a noninvasive treatment for TTNS that replaces fine needles with surface electrodes. In the study by Sonmez et al., Ramírez-García et al., and Zonić-Imamović et al., PTNS and TTNS had similar clinical effects [31, 33, 34], but the results of this network meta-analysis showed that there was no statistically significant difference between PTNS and TTNS in the improvement of the daytime micturition frequency, but the probability ranking of RANK was better for PTNS than TTNS. PTNS was better than TTNS in improving nocturia frequency, possibly because fine needle aspiration enables electrical stimulation to act directly on the nerve and the effect is more precise; however, PTNS has more adverse reactions than TTNS [31, 32].

The therapies included in this network meta-analysis all improved symptoms of OAB, with therapy durations ranging from 8 to 12 weeks; however, according to existing studies, most of these improvements were time-limited, and symptoms of OAB gradually worsened as treatment was stopped for longer [25, 32, 35, 36]. The effective control of OAB in the long term is worthy of further study, including physical and rehabilitation therapies, such as BT, behavioral therapy, PFMT, and TTNS, which can be independently treated at home [31, 35, 37], but PTNS, BF, and ES, etc., need to be carried out by professionals in medical institutions with professional equipment [4, 21, 30, 33]. PTNS, BT + ES, and BT + BF + ES can be the priority therapy when circumstances permit.

The strengths of our network meta-analysis include the following: (1) minimizing publication bias, we searched all domains using keyword combinations; (2) faced with different research methods, we only included randomized controlled trials; (3) the results of the study combined direct and indirect comparisons; (4) Bayesian network meta-analysis was conducted [38] to compare the treatment effects of different physical and rehabilitation therapies on OAB; and (5) posterior probabilities and SUCRA were used to distinguish differences between the 12 physiotherapy and rehabilitation therapies. However, these are some limitations to this network meta-analysis: (1) the number of included studies and patients was small; (2) characteristics vary among different studies, which may have a potential impact on the results of the research; and (3) many symptom evaluation indicators and evaluation methods of OAB lack a standardized and unified evaluation system; (4) only two representative indicators of daytime micturition frequency and nocturia frequency were selected; (5) the duration of the intervention included in the studies was short; and (6) there was a lack of assessment of the long-term effect of improvement in OAB symptoms. Therefore, larger randomized controlled trials that include assessments of long-term improvement in OAB symptoms are needed to enrich the conclusions of this network meta-analysis.

## 6. Conclusions

Different physical and rehabilitation therapies can improve the symptoms of OAB. Each institution should choose physical and rehabilitation therapies according to its own setup and personnel base. PTNS, BT + ES, and BT + BF + ES can be considered priority therapies when the situation permits.

## Figures and Tables

**Table 1:** Characteristics of enrolled studies.

Authors	Years	Treatments	Samples	Outcomes
Bellette	2009	PTNS Placebo	21 16	Daytime micturition frequency Nocturia frequency

Bykoviene	2018	PFMT PFMT + TTNS Placebo	20 19 22	Daytime micturition frequency Nocturia frequency

Firinci	2020	BT BT + BF BT + ES BT + BF + ES	17 16 17 16	Daytime micturition frequency Nocturia frequency

Hargreav	2021	Placebo Acupuncture	13 16	Daytime micturition frequency Nocturia frequency

Jacomo	2020	TTNS SNS	25 25	Daytime micturition frequency Nocturia frequency

Martin-Garcia	2019	PTNS TTNS	12 12	Daytime micturition frequency

O'Reilly	2008	SNS Placebo	33 30	Daytime micturition frequency

Scaldazza	2017	PFMT + ES PTNS	30 30	Daytime micturition frequency Nocturia frequency

Sonmez	2022	BT PTNS TTNS	19 19 20	Daytime micturition frequency Nocturia frequency

Tezer	2022	BT BT + MStim	36 35	Daytime micturition frequency Nocturia frequency

Yildiz	2021	BT BT + ES	29 29	Daytime micturition frequency Nocturia frequency

Zonić-Imamović	2021	TTNS PTNS	30 30	Daytime micturition frequency nocturia frequency

PTNS, percutaneous tibial nerve stimulation; PFMT, pelvic floor muscle training; TTNS, transcutaneous stimulation of the tibial nerve; ES, electrical stimulation; BT, bladder training; BF, biofeedback; SNS, sacral nerve stimulation; and MStim, magnetic stimulation.

## Data Availability

All data and codes are available from the corresponding author upon request.
